# Baicalin Inhibits EMT through PDK1/AKT Signaling in Human Nonsmall Cell Lung Cancer

**DOI:** 10.1155/2021/4391581

**Published:** 2021-11-25

**Authors:** Jia Chen, Cheng-Bo Yuan, Bo Yang, Xuan Zhou

**Affiliations:** ^1^Affiliated Hospital of Changchun University of Traditional Chinese Medicine, Changchun, Jilin, China; ^2^Department of Intensive Care Medicine, Affiliated Hospital of Jining Medical University, Jining, Shandong, China

## Abstract

**Background:**

Baicalin is a naturally occurring compound with anticancer, antioxidant, and anti-inflammatory properties. However, the mechanism underlying its anticancer activity on nonsmall cell lung cancer (NSCLC) remains unclear.

**Methods:**

The effects of baicalin on the progression and metastasis of experimental NSCLC cell lines were studied *in vitro* and *in vivo*. Wound-healing and transwell assays were performed to evaluate the potency of baicalin and the motility and migration ability of NCI-H460 cells. Immunofluorescence assay, western blot assay, and immunohistochemistry test were conducted to investigate the inhibiting effect of baicalin on the epithelial-mesenchymal transition (EMT) of NSCLC.

**Results:**

Baicalin inhibited the proliferation and migration of NCI-H446 human NSCLC cells in a dose-dependent manner, reduced the expression levels of phospho-3-phosphoinositide-dependent protein kinase 1 (p-PDK1) and phosphor-serine/threonine-protein kinase (p-AKT), reversed the levels of EMT markers, and inhibited the migration of NSCLC cells.

**Conclusions:**

Baicalin impedes EMT by inhibiting the PDK1/AKT pathway in human NSCLC and thus may be an effective alternative treatment for carcinoma and a new candidate antimetastasis drug.

## 1. Introduction

Lung cancer is a common solid cancer with high cancer-related mortality worldwide [1] accounting for 1.6 million deaths each year [2]. Among all cancers, lung cancer has the worst prognosis [3]. Approximately 70% of patients with lung cancer have other metastatic diseases upon detection. Nonsmall cell lung cancer (NSCLC) is a histological subtype of lung cancer and accounts for 85% of its occurrence in the United States [4]. NSCLCs are of epithelial origin and include squamous cell carcinomas and adenocarcinomas. Surgical resection and traditional chemotherapy are most commonly used to treat early stage or advanced NSCLC, but these methods cannot achieve good results [5, 6]. Therefore, finding safe, effective, and affordable therapeutics for this disease is urgent.

Baicalin is a flavonoid compound derived from *Scutellaria baicalensis* Georgi or other medicinal herbs and has various pharmacological effects, including antioxidant, anti-inflammation, and antitumor activities [7–10]. This compound inhibits the metastasis of highly aggressive breast cancer [11] and suppresses the proliferation, migration, and invasion of human glioblastoma cells [12]. Baicalin can also induce cancer cell apoptosis *in vitro* and suppress tumor growth *in vivo* [13]. However, the mechanism underlying its anticancer activity on NSCLC remains unclear.

NSCLC is highly susceptible to invasion and metastasis, which are closely related to the occurrence of EMT [14, 15], the transformation of epithelial cells to mesenchymal cells. EMT is first noted during embryogenesis, and the developmental program of mesenchymal cells promotes cell invasion and proliferation [16]. Such plasticity may reverse mesenchymal-epithelial transition, thereby accelerating the formation of secondary clone sites [17]. EMT can also upregulate the vimentin and N-cadherin expression levels and downregulate the expression levels of epithelial biomarkers, such as E-cadherin and claudin.

Phosphoinositide-dependent kinase-1 (PDK1) is one of the AGC kinase family members, a serine-threonine kinase, and a recently discovered signaling hub for the migration and invasion of tumor cells [18]. Serine/threonine kinase (AKT) is a member of the SGK family of kinases, and its phosphorylation is regulated by PDK1, an upstream kinase of AKT [19]. The activation/inhibition of the PDK1/AKT signaling pathway regulates the proliferation, invasion, metastasis, and survival of human cancer cells [20]. This pathway is also related to many types of cancer and acts as a major signaling pathway of the oncogenesis in tumor that is active in pancreatic cancers [21], breast cancer [22], and hepatocellular carcinoma [23]. Therefore, this pathway is a possible therapeutic target in patients with cancer. Our research showed that baicalin can suppress tumor progression by targeting the PDK1/AKT signaling pathway to inhibit EMT on NSCLC.

## 2. Results

### 2.1. Baicalin Inhibited NSCLC Proliferation

**Figure 1:**
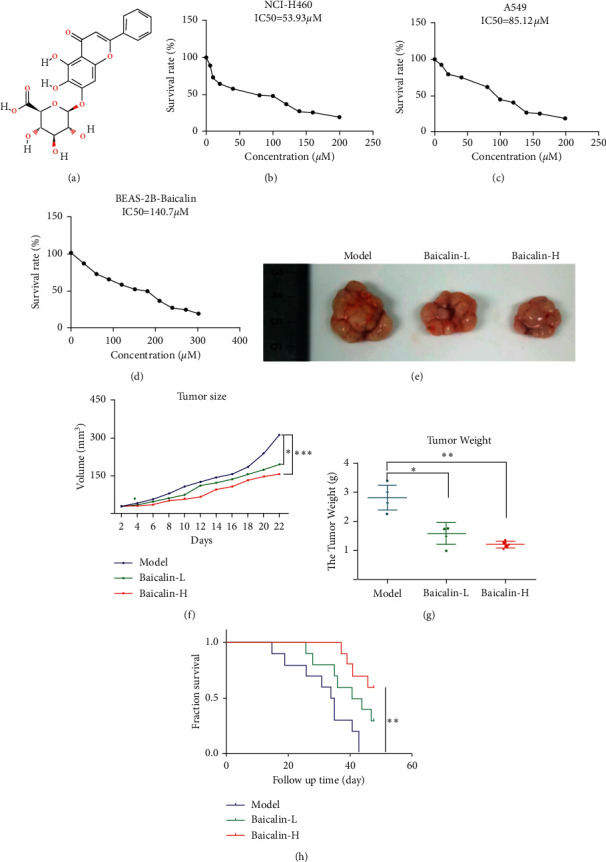
Baicalin inhibited NSCLC proliferation. (a) Molecular formula of baicalin. (b–d) NCI-H460, A549, and BEAS-2B cells were treated with baicalin. Cell viability was tested by MTT assay. (e) Scheme of the tumor model of nude mice with or without baicalin treatment. (f, g) Effects of baicalin on the tumor weights and tumor volume of NSCLC tumor mice. (h) Effects of baicalin on survival in NSCLC cell-bearing nude mice.

The molecular formula for baicalin is shown in Figure 1(a), and its effects on cell activity were determined by MTT assay (treated for 48 h). Figures 1(b)–1(d) show that baicalin dose-dependently inhibited the proliferation of the lung cancer cells NCI-H460 and A549 and normal cells BEAS-2B with IC50 values of 53.93, 85.12, and 140.7 *μ*M, respectively. This finding suggests the high inhibitory potency of this compound. Animal experiments were designed to investigate whether baicalin remarkably affects the proliferation of lung cancer in vivo. Tumor growth was suppressed in nude mice with baicalin treatment compared with that in the model groups (Figure 1(e)). Tumor weight and volume decreased in low (baicalin-L) and high dose (baicalin-H) treatment groups (Figures 1(f) and 1(g)). The survival time of mice in baicalin-L and baicalin-H groups was significantly prolonged (Figure 1(h)).

### 2.2. Baicalin Suppressed NSCLC Cell Migration, Invasion, and Stemness

**Figure 2:**
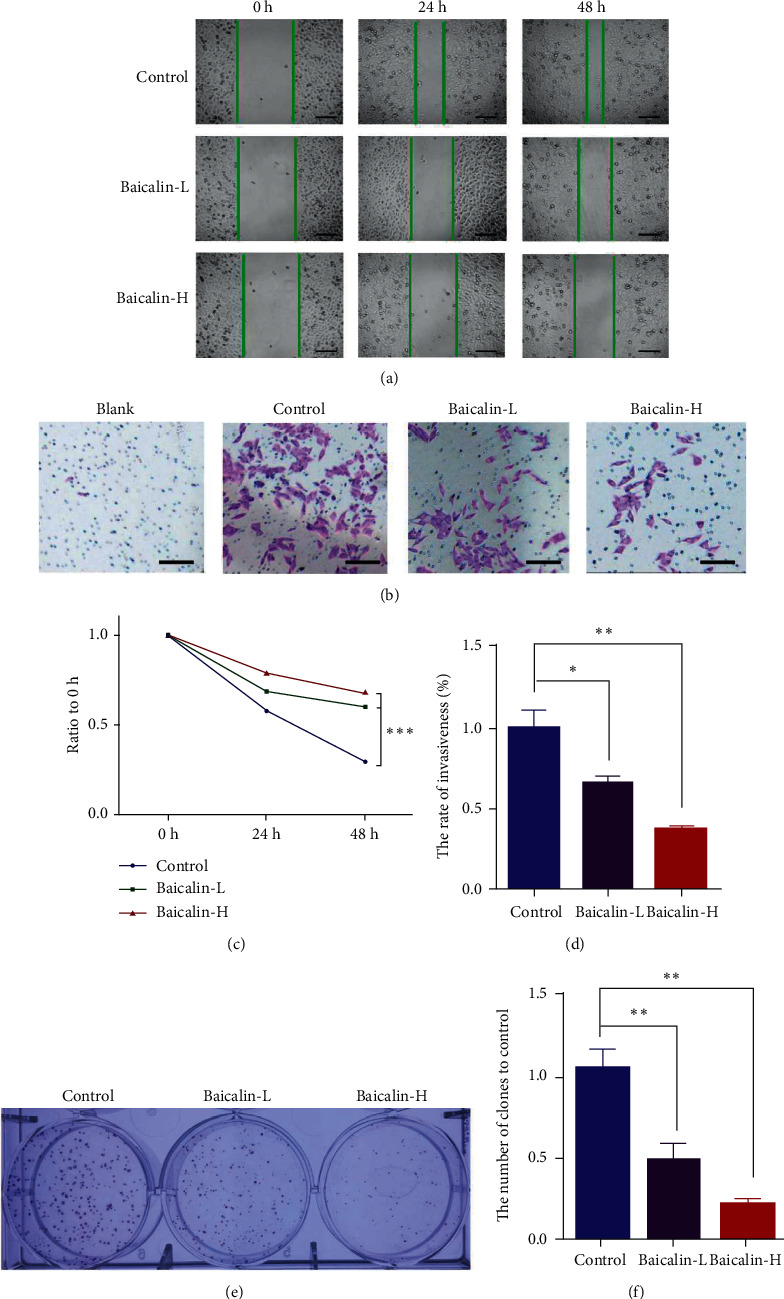
Baicalin suppressed NSCLC cell migration, invasion, and stemness. (a, c) Cell migration was measured after treatment with baicalin for 24 and 48 h. (b, d) Representative images and statistical results of transwell cell invasion assays were obtained at 200x magnification. (e, f) Representative images and statistical results of clone formation of NCI-H460 cells stained with crystal violet.

Wound-healing and transwell assays were performed to evaluate the potency of baicalin and the motility and migration ability of NCI-H460 cells. The cancer cells were exposed to increase the baicalin doses up to 30 *μ*M, and the migration abilities of NCI-H460 cells were significantly and dose-dependently inhibited (Figures 2(a) and 2(c)). Transwell assays also showed that baicalin inhibited cell invasion (Figures 2(b) and 2(d)). Plate colony formation assay was designed to study whether baicalin can suppress the colony formation of NCI-H460. Figures 2(e) and 2(f) showed that 15 and 30 *μ*M baicalin dose-dependently suppressed the stemness of NCI-H460 cells.

### 2.3. Baicalin Reversed EMT of NSCLC

**Figure 3:**
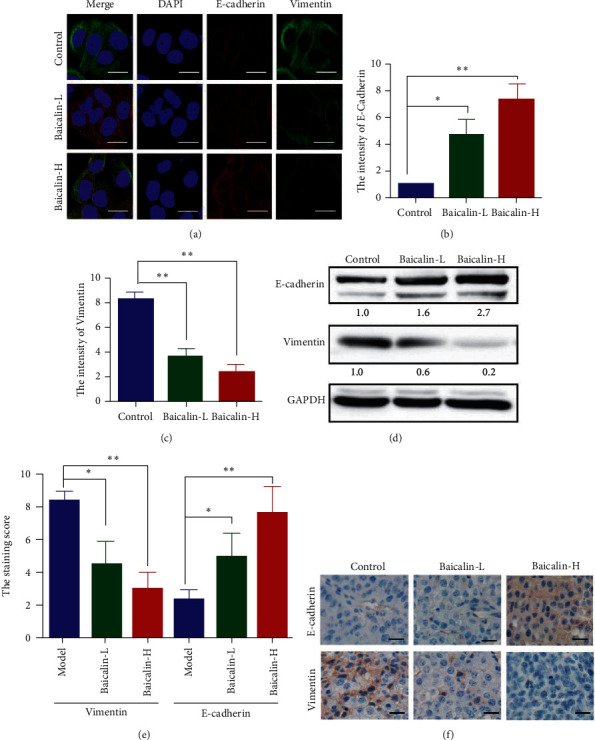
Baicalin reversed EMT of NSCLC. (a–c) Double immunofluorescence staining for E-cadherin and vimentin after treating with baicalin. (d) Protein expression levels of E-cadherin and vimentin after treatment with baicalin. (e, f) The E-cadherin and vimentin expression levels in the tumor tissues of subcutaneously transplanted tumor mice.

Immunofluorescence assay, western blot assay, and immunohistochemistry assay were conducted to investigate the inhibiting effect of baicalin on the EMT of NSCLC. NCI-H460 cells were treated with baicalin at different concentrations, and the expression levels of EMT markers were observed by laser confocal microscopy. Baicalin increased the expression of epithelial marker E-cadherin but decreased that of mesenchymal marker vimentin in a dose-dependent manner (Figures 3(a)–3(c)). Western blot results showed similar trends on NCI-H460 cells with baicalin treatment (Figure 3(d)). Similar results were also observed in subcutaneous xenotransplanted tumor as shown in Figures 3(e) and 3(f). These results indicated that baicalin effectively inhibited the EMT of NSCLC.

### 2.4. Baicalin Inhibited PDK1/AKT Signaling Pathway in NSCLC

**Figure 4:**
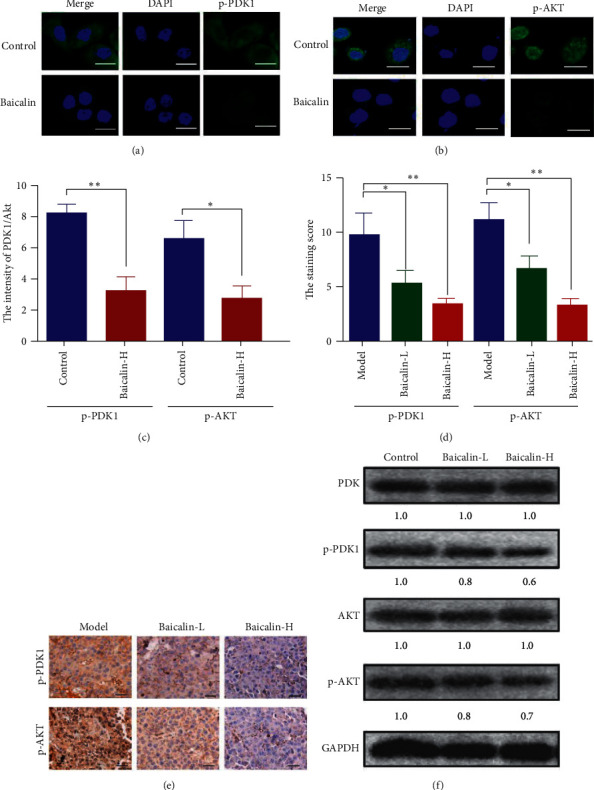
Baicalin inhibited the PDK1/AKT signaling pathway in NSCLC. (a–c) Immunofluorescence staining for p-PDK1 and p-AKT after treating with baicalin. (d) Protein expression levels of p-PDK1 and p-AKT after treating with baicalin. (e, f) The p-PDK1 and p-AKT expression levels in tumor tissues of subcutaneously transplanted tumor mice.

The p-PDK1 and p-AKT levels in tumor were tested by immunofluorescence assay using whole cell lysates from NCI-H460 cells treated with baicalin for 48 h. The p-PDK1 and p-AKT level decreased when treated with baicalin (Figure 4(a)–4(c)). Similar results were also observed in subcutaneous xenotransplanted tumor (Figures 4(d) and 4(e)) and western blot results (Figure 4(f)).

## 3. Discussion

NSCLC has been reported in approximately four-fifths of patients with lung cancer [24]. This subtype has a poor prognosis with chemotherapy as first-line treatment [4], and 90% of NSCLC deaths are related to metastasis [25]. In this study, the effect of 48 h of treatment with baicalin on cell viability in NSCLC cell lines was analyzed using MTT assay. Baicalin dose-dependently reduced cell viability. At 30 *μ*M, this compound considerably inhibited migration and clone formation in NSCLC cell lines.

Baicalin has low cytotoxicity and antimetastatic activity in various cancer cells; however, the mechanism underlying this effect remains unclear [26]. Baicalin is a flavonoid compound mainly derived from scutellaria baicalensis, which contains natural active flavone constituents, such as baicalein, baicalin, wogonin, wogonoside, and oroxylin A. These molecules all have low cytotoxicity and significant anticancer effect in various cancer cells. The metastatic processes start at the tumor cells that develop their invasive properties during EMT [27]. Over the past decade, the importance of EMT during the progression in various carcinomas, especially NSCLC, has received increasing attention [28, 29]. The expression levels of EMT markers change during this process and results in the rearrangements of cellular cytoskeletal that favor metastasis [30].

Our results showed that baicalin inhibits the EMT of NSCLC in vitro and in vivo. E-cadherin and vimentin (EMT biomarkers) mediate cell adhesion. E-cadherin downregulation is necessary to provide metastatic ability to NSCLC cells. Vimentin, which is an intermediate filament protein, forms the cytoskeleton along with microtubules and actin filaments. In this study, baicalin increased E-cadherin and decreased vimentin levels in NSCLC.

PDK1 pathway is highly activated in NSCLC cells and a potential target for tumor therapy [31]. The inhibition of PDK1 expression level can repress tumor progression and metastasis [32]. PDK1 is an important regulatory factor in multiple extracellular stimuli-mediated pathways and plays an important role in AKT activation [33]. Our study revealed that baicalin significantly inhibits the PDK1/AKT pathway.

However, this study did not involve in the clinical experimental data of baicalin. Moreover, reports are rare about the clinical studies of baicalin. In the future study, we plan to allow advanced lung cancer patients to normal treatment and the baicalin tablets at the same time, a prescription drug for acute and chronic hepatitis, and to evaluate the efficacy of the drug.

## 4. Conclusion

In conclusion, baicalin represses cancer invasion by inhibiting EMT through the PDK1/AKT signaling pathway. Our results provide a new mechanistic basis for the therapeutic application of baicalin in patients with NSCLC. Baicalin may be an effective alternative treatment for persistent carcinoma and a new candidate antimetastasis drug.

## 5. Materials and Methods

### 5.1. Cell Culture and Antibodies

A549, NCI-H460, and BEAS-2B cells were purchased from KeyGEN BioTECH (Nanjing, China). A549 cells were maintained in RPMI 1640, and NCI-H460 cells were cultured in DMEM complete medium with 10% FBS. All cell lines were cultured at 37°C and 5% CO_2_ in an incubator. p-PDK S241 (Abcam, ab278567, 1 : 1000), p-AKT S473 (affinity, AF0016, 1 : 1000), E-cadherin (Abcam, ab40772, 1 : 1000), and vimentin (affinity, BF8006, 1 : 1000) were used for Western blot and immunofluorescence assay.

### 5.2. Cell Viability Assay

NCI-H460 cells were seeded into a 96-well cell culture plate, incubated in 5% CO_2_ air at 37°C overnight and then cultured on complete media with different baicalin concentrations for 48 h. Cell viability was monitored by MTT assay (MTT, Promega, G4000). Approximately 20 *μ*L of 5 mg/mL MTT was added to a 96-well cell culture plate and incubated at a constant temperature incubator for 4–6 h. In the medium containing MTT, 100 *μ*L of DMSO was added per well to dissolve the formazan crystals of the cells. The 96-well plate was placed in the microplate reader to detect the OD value, and the results were analyzed by GraphPad prism. All experiments were repeated at least three times.

### 5.3. Wound Healing Assay

Baicalin suppressing cell migration was performed by wound-healing assay. Cells were seeded in a 24-well plate and cultured to 100% confluent monolayers. A scratch wound was made with 100 *μ*L of imported tips, and the cells were then washed with 1× PBS and incubated with different baicalin concentrations (0, 15, and 30 *μ*M) for 48 h. The images of the scratch wound after 0, 24, and 48 h were captured using an optical microscope (Nikon, Japan). Area for wound was computed using the Nikon Elements Basic Research Software analysis tools, and the data of the wound were analyzed by *t*-test using GraphPad Prism 7 software. Data were analyzed at least three times.

### 5.4. Transwell Assays

Cell invasion ability was assessed through transwell assays. The chamber below was suspended in 300 *μ*L of medium containing different concentrations of baicalin without FBS, and the cancer cells were seeded into matrigel-coated upper wells covered with 8 *μ*m pore-sized polyethylene terephthalate filter membrane. Approximately 500 *μ*L of medium containing 10% FBS was placed in the lower chamber. Cotton swabs were used to remove the cells on the upper surface of the filter after 48 h of incubation. The cells were then fixed with 4% paraformaldehyde for 20 min. Afterward, these cells were stained with 0.1% crystal violet for 10 min. Invading cells were imaged by an inverted microscope (Nikon, Japan) at 100x magnification, and the cell number was manually counted.

### 5.5. Flat Plate Clone Formation Assay

The NCI-H460 cells were digested, seeded into a 6-well plate, treated with different concentrations of baicalin (0, 15, and 30 *μ*M), and continuously cultured for 48 h. The baicalin-containing medium was removed and washed with PBS two times, and then the cells were cultured in DMEM at 37°C for 10 days. Finally, the cells were fixed with 95% methanol (4%, 15 min, 37°C) and stained with crystal violet for 10 min. Colony numbers with >50 cells were analyzed with an Olympus digital camera. The experiments were repeated at least three times.

### 5.6. Western Blot Assay

NCI-H460 cells were treated with or without baicalin and collected using cell lysis kit after 48 h of treatment for the preparation of total cellular lysates. BCA protein assay kits were used to assess the PDK1 and AKT concentrations. Whole protein (30 *μ*g/lane) was separated using SDS-polyacrylamide gel electrophoresis and then transferred into the PVDF membranes. Nonspecific sites on the PVDF membrane were blocked with 5% skimmed milk powder in TBST for 1 h. The PVDF membrane was incubated with primary antibodies against PDK1, AKT, E-cadherin, vimentin, GAPDH, and tubulin at 4°C overnight and then washed with PBST three times. The protein on PVDF membrane was studied by the ECL method.

### 5.7. Immunofluorescence Assay

The cells were harvested and seeded on glass coverslips in 24-well plates for 24 h. When the cells grew to 50% confluence, they were exposed to different baicalin concentrations (0, 15, or 30 *μ*M) for 48 h. Treated and untreated cells were washed with PBS, fixed in 3.7% formaldehyde for 20 min, and permeabilized with 0.2% Triton X-100 in PBS. The slides were washed with PBS and incubated with 5% goat serum in PBS for 2 h and then incubated with primary antibodies anti-E-cadherin, antivimentin, anti-PDK1, or anti-AKT (1 : 100, 4 °C, overnight). Then, the cells were washed with PBS and incubated with Alexa Fluor 488 goat anti-mouse IgG or Alexa Fluor 594 goat anti-rabbit IgG to protect from light (1 : 200, 1 h, 37°C). Coverslips were washed and used to cover upside down on a slide containing DAPI. Pictures were taken with a confocal microscope (N-STORM, Nikon, Tokyo, Japan).

### 5.8. Animal Studies

BALB/c nude mice weighing 18–20 g were obtained from the Animal Center Academy of the Military Medical Science (Beijing, China). All animals were bred under specific pathogen-free conditions. The mice were fed a standard laboratory diet and given water ad libitum to the most comfortable living environment. All procedures were approved based on guidelines of the Animal Ethics Committee of the Changchun University of Traditional Chinese.

Nude mice were infected with 1 × 10^6^ NCI-H460 cells and divided into 3 groups randomly (5 mice per group), including animal hair, activity level, and diet and drinking conditions. Tumor volume was measured every day. A total of 0.9% NaCl, 40 mg/kg baicalin-L, and 60 mg/kg baicalin-H were administered daily by oral gavage for 3 weeks according to the indicated body weight of the mice. The survival status of the remaining mice was monitored continuously. On the 17th day after tumor bearing, some mice in the model group stopped free crawling, became sluggish and lethargic, loose fur stood up, decreased their activities, and some animals showed sideways lying and then had weak limbs. Then, the mice were euthanized with 150 mg/kg pentobarbital sodium at the 22th day. The mice in the administration groups were significantly more active than those in the model group. Lastly, the mice were sacrificed in a humanitarian manner, and tumor tissues were fixed with paraformaldehyde. Another 30 mice were allocated randomly to 3 groups as described above (10 mice per group) to measure survival rates until their natural death. At last, we checked whether the animal's heart is beating to verify the death of the mice. In our experiment, the maximum diameter of mice can reach 300 mm^3^ until their natural death based on our prior observation in non-small lung cancer mouse model. The survival time of every mouse was recorded and lasted for 48 days.

### 5.9. Immunohistochemistry (IHC)

Tissue section was deparaffinized and rehydrated through ethanol. The primary antibodies and dilution ratios are listed as follows: p-Akt S473 (1 : 100), p-PDK1 (1 : 50), E-Cadherin (1 : 100), and vimentin (1 : 50); PBS replaced the first antibody as the negative control. A four-tiered scale was used to grade staining intensity in tumor cells. The percentages of positive cells were divided into five classes based on the percentage of tumor cells stained.

This study was approved by the Institutional Animal Care and Use Committee(IACUC) at Changchun University of Traditional Chinese in accordance with national and international guidelines. All animals in this experiment were very well taken care of. The permit number is ST-2018005.

### 5.10. Statistical Analysis

All data were from biological triplicate experiments and presented with error bar as mean ± S.D. Two groups of data were analyzed by two-tailed unpaired Student's *t*-test. Two-ANOVA (Tukey's multiple comparison test) was used to compare multiple groups of data. All statistical testing results were processed with SPSS 17.0 software (^*∗*^*P* < 0.05 and  ^∗∗^*P* < 0.01).[18]

## Data Availability

The data used to support the findings of this study are available from the corresponding author upon request.
